# Supplementary material for: JAC4 Protects from X-ray Radiation-Induced Intestinal Injury by JWA-Mediated Anti-Oxidation/Inflammation Signaling
Source: Antioxidants (Basel). 2022 May 27;11(6):1067. doi: 10.3390/antiox11061067 (PMC9220415; doi:10.3390/antiox11061067)
Supplement: Supplementary file 1 [file antioxidants-11-01067-s001.zip › antioxidants-1719696-supplementary.pdf]

antioxidants

MDPI

Supplementary Materials: JAC4 Protects from X-Ray Radiation-Induced Intestinal Injury by JWA-Mediated Anti-Oxidation/Inflammation Signaling

Yan Zhou [1,2,†], Jingwen Liu [1,2,†], Xiong Li [1,2], Luman Wang [1,2], Lirong Hu [3], Aiping Li [1,2] and Jianwei Zhou [1,2,*]

1 Department of Molecular Cell Biology & Toxicology, Center for Global Health, School of Public Health, Nanjing Medical University, 101 Longmian Avenue, Nanjing 211166, China; zhouyan_1223@163.com (Y.Z.); anglemuyan@foxmail.com (J.L.); hllixiong@163.com (X.L.); hdlxdlwlm@126.com (L.W.); liaiping@njmu.edu.cn (A.L.)
2 The Key Laboratory of Modern Toxicology, Ministry of Education, School of Public Health, Nanjing Medical University, 101 Longmian Avenue, Nanjing 211166, China
3 State Key Laboratory of Translational Medicine and Innovative Drug Development, Jiangsu Simcere Pharmaceutical Co., Ltd., Nanjing 210042, China; hulirong@simcere.com
* Correspondence: jwzhou@njmu.edu.cn
† These authors contributed equally to this work.

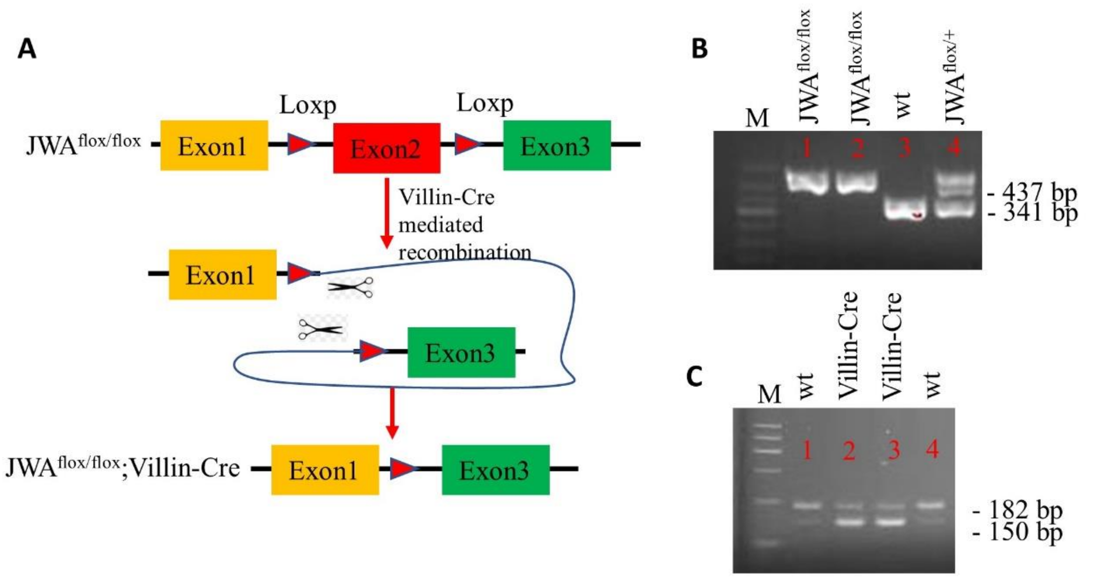

Figure S1. Construction and genotype verification of the intestinal epithelial JWA deletion mouse. (**A**) The construction of JWA$^{flox/flox}$;Villin-Cre; (**B**)Genotyping of JWA$^{flox/flox}$ mice by PCR; (**C**) Genotyping of Villin-Cre mice by PCR.

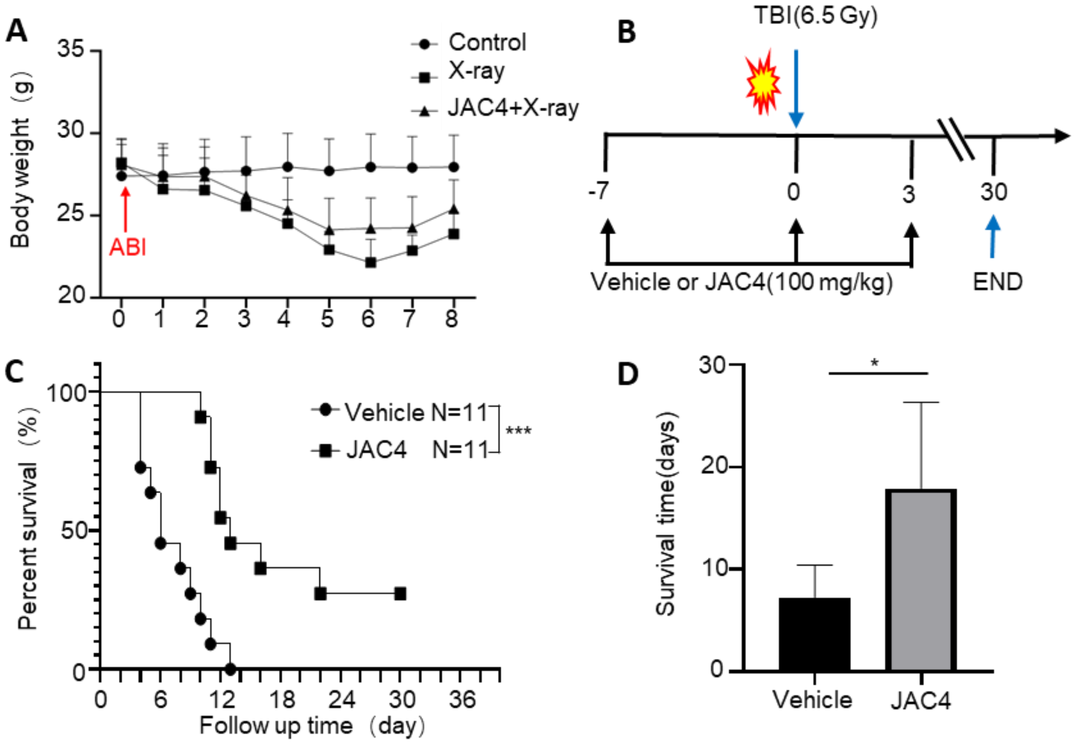

Figure S2. JAC4 improved the mice weight loss after ABI and the survival rate of mice after TBI. (**A**) The body weight of the mice after ABI; (**B**) C57BL/6 mice (n = 11) were administered JAC4 (100 mg/kg) or vehicle by gavage for seven days, on the seventh day mice were exposed to total body radiation (6.5 Gy); JAC4 treatment was continued for additional three days, then monitored for 30 days; (**C**) Kaplan-Meier survival analysis of mice exposed to 6.5 Gy TBI, ***$P < 0.001$, n = 11; (**D**) The average survival time of the two groups of mice, *$P < 0.05$.

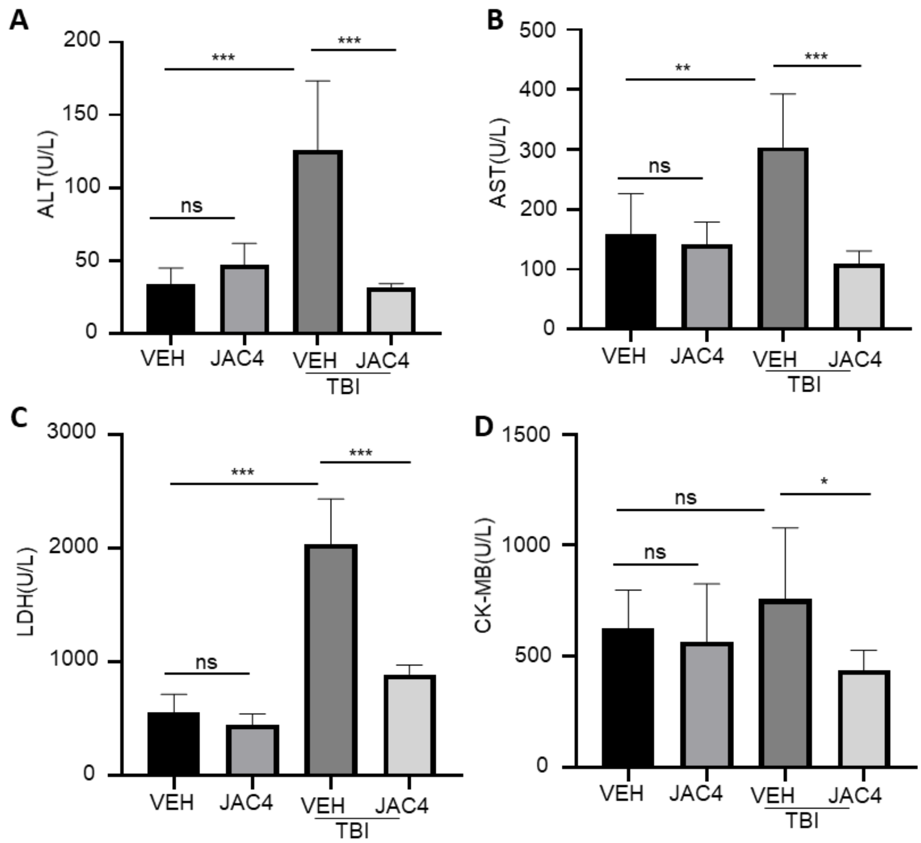

Figure S3. JAC4 reduced the organ toxicity of heart and liver induced by TBI. Serum contents of ALT(**A**), AST(**B**), LDH(**C**) and CK-MB(**D**) were determined on 4th day after ABI (n=8); the results were expressed as mean ± SEM, n=6; *$P < 0.05$; **$P < 0.01$; ***$P < 0.001$; ns: $P > 0.05$.

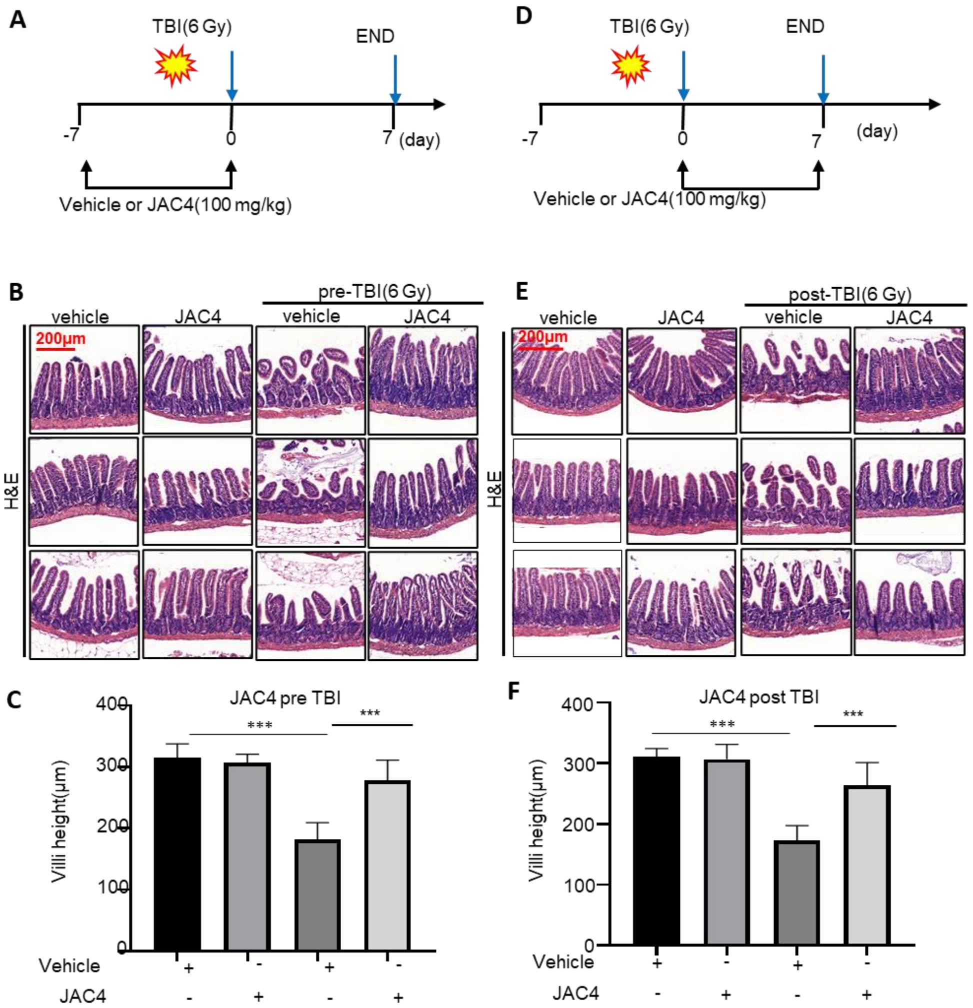

Figure S4. Preventive or therapeutic administration of JAC4 reduced the damage of small intestinal epithelium after TBI. (**A**) Mice (n = 6) were administered JAC4 (100 mg/kg) or vehicle by gavage for seven days, on the seventh day mice were exposed to TBI (6 Gy); and small intestine tissues were collected for (**B**)H&E staining of small intestine after seven days of TBI; (**C**) Villus height of JAC4 prophylactic administration model; (**D**) Mice (n = 6) were administered JAC4(100 mg/kg) or vehicle by gavage for seven days after TBI (6 Gy), then small intestine tissues were collected for (**E**)H&E staining of small intestine after seven days; (**F**) Villus height of JAC4 therapeutic administration model; the results are presented as mean ± SD, n = 6; $^{***}P < 0.001$; ns: $P > 0.05$.

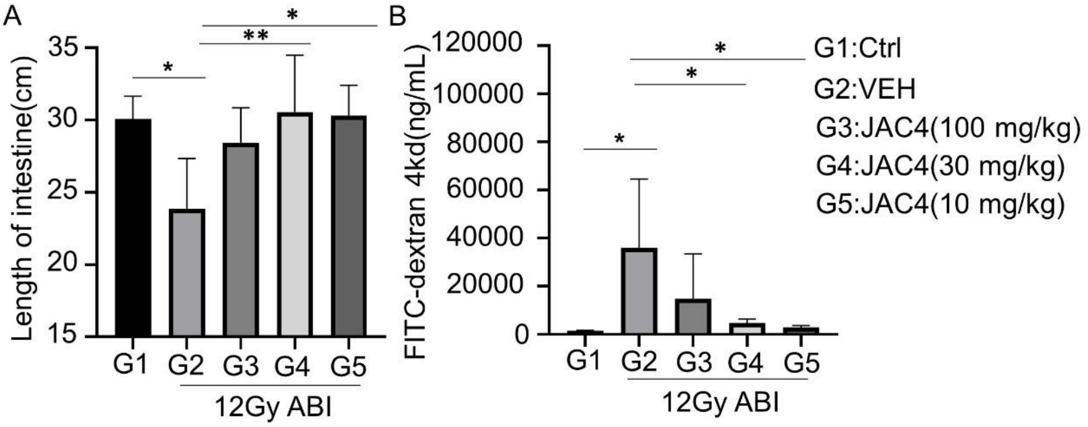

Figure S5. Different doses of JAC4(10/30/100 mg/kg) reduced the damage of small intestinal epithelium after ABI. C57BL/6 mice were treated with either vehicle or JAC4 (10/30/100 mg/kg/day) 7 days before-exposure and 4 days post-exposure of 12 Gy ABI and analyzed the length of small intestine and FD4 concentration at 4 days after ABI. (**A**) quantitative statistics of small intestine length; (**B**) The concentration of FITC-dextran in the plasma; the results are presented as mean ± SD, n = 5; $^*P < 0.05$;$^{**}P < 0.01$; ns: $P > 0.05$.

Table S1. Primer for genotypic identification of mice.

**primer**	**Primer sequences**
JWA-Loxp	Forward：5′-CCACTGTTTCCTCTGTTG-3′
	Reverse：5′-GTGAAAACCACTGAGAACC-3′
Villin-Cre	Common Forward：5′-GCCTTCTCCTCTAGGCTCGT-3′
	Wild type Reverse：5′-TATAGGGCAGAGCTGGAGGA-3′
	Mutant Reverse：5′-AGGCAAATTTTGGTGTACGG-3′
